# On spatial pattern of concentration distribution for Taylor dispersion process

**DOI:** 10.1038/srep20556

**Published:** 2016-02-12

**Authors:** Zi Wu, Xudong Fu, Guangqian Wang

**Affiliations:** 1 State Key Laboratory of Hydroscience and Engineering; Department of Hydraulic Engineering, Tsinghua University, Beijing 100084, China; 2 National Center for Earth-surface Dynamics and St. Anthony Falls Laboratory, University of Minnesota, Minneapolis, Minnesota 55414, USA

## Abstract

Taylor dispersion is a key concept in many fields. In the present paper, we characterize the pattern of the complete spatial concentration distribution for laminar tube flow; the obtained simple description is shown to represent the nature of Taylor dispersion. Importantly, we find that during the approach to the longitudinal normality of the transverse mean concentration at the time scale of *R*^*2*^/*D* (*R* is the tube radius and *D* is the molecular diffusivity), the solute concentration becomes uniformly distributed across a family of invariant curved transverse surfaces instead of the flat cross-sections in the traditional view. The family of curved surfaces is analytically determined, and a transformation is devised for the previously obtained analytical solution to discuss the decay of the concentration difference across the curved surfaces. The approach to a uniform concentration across the flat cross-sections to the same degree (~3% by concentration difference percentage), achieved at a time-scale of 100 *R*^*2*^/*D*, is shown to be the natural consequence of the longitudinal separation of the concentration contours on the curved surfaces.

Studies of Taylor dispersion^1^ were originally aimed to understand the transport of a soluble salt in blood flows and to develop a means for measuring molecular diffusivity, which remains a standard method even nowadays^2^. Taylor dispersion is of fundamental importance and has been widely applied in the fields of environmental science and engineering, biomedical engineering, chemical engineering, and so on^3^,^4^,^5^,^6^,^7^,^8^,^9^,^10^,^11^,^12^,^13^. In the study of scalar transport in laminar tube flows of G. I. Taylor^1^, in a dimensionless form, Taylor dispersion describes the transverse mean concentration 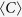 governed by a diffusion equation in a constantly moving coordinate system:


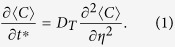


Here, the angle brackets 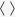 define the transverse average for any quantity *f*:


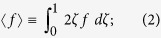



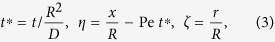


where *t* is the time, *R* is the radius of the tube, *D* is the molecular diffusivity, *x* is the longitudinal coordinate, *r* is the radial coordinate, and Pe is the Peclet number:


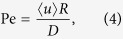


where *u* is the flow velocity in the tube and is a function of the radial coordinate *r*. In Eq. (1), the Taylor dispersivity 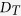 is an effective diffusion coefficient and has been analytically determined to be 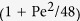
1,14.

Taylor dispersion is primarily a theory for the transverse mean concentration, as reflected by Eq. (1). Because of Taylor’s initiative, numerous efforts have been made to explore the evolution of the mean concentration^3^,^9^,^15^,^16^,^17^,^18^,^19^,^20^,^21^,^22^,^23^,^24^,^25^,^26^,^27^. Based on many studies, fundamental issues were raised, for example, one of the most important issues is the question of the validity of the Taylor dispersion model. Chatwin^18^ systematically addressed the issue and provided a well-known estimation for the time scale. However, the mean concentration distribution provides only some of the information that is needed to understand the physical process of concentration transport, and less effort has been made with regard to study the transverse concentration distribution and evolution^9^,^28^. This situation is the exact starting point of the previous work of Wu and Chen^9^, who addressed the approach to uniformity of the concentration distribution referring to the flat cross-sections, which belongs to another fundamental issue.

Despite having been studied for over sixty years with fruitful progress, some fundamental issue about the physical process of scalar transport in Taylor dispersion regime remains unclear. *The pattern of the complete spatial concentration distribution has not been suitably characterized yet*, although different methods have been proposed that can be applied for the transverse distribution^9^,^18^,^29^,^30^. Until very recently, some researchers believed that when Eq. (1) becomes valid for the mean concentration, the concentration difference should be small over the cross-section of the tube^1^,^17^,^19^,^31^. It was believed that the transitions, for the mean concentration to approach normality^18^ and for the transverse concentration to approach uniformity^9^, should be regarded as one basic process^17^ and can be described by the time scale of 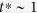. This statement has turned out to be not true^9^: the transverse concentration difference remains significant for a very long period of time, which is characterized by the time scale of 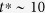. However, using only observations with reference to the flat cross-sections in the tube, by the acquired information it is too complicated to draw any sound conclusion for the transverse concentration of distribution patterns^9^. Most importantly, the interrelations between the proposed two time scales, corresponding to the two basic physical processes mentioned above, are still unknown.

This paper is organized as follows to properly describe the complicated spatial pattern of the concentration distribution during Taylor dispersion. We first present a family of invariant curved transverse surfaces, across which the solute concentration will be uniformly distributed. Next, we show how the surfaces can be analytically determined by a first estimation. Subsequently, the physical insight of this finding is discussed as a sign of the transition to Taylor dispersion regime, with a transformation defined for evaluating the degree of uniformity across the surfaces. In part 3, we verify our findings using some analytical and numerical results. Conclusions are provided in the last section.

## Analytical Considerations

### A family of invariant curved transverse surfaces

The key contribution of this paper is the characterization of the multi-dimensional concentration distribution pattern during Taylor dispersion process. Specifically, we find that after the establishment of Taylor dispersion at the time scale of 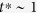, *the solute concentration will be uniformly distributed across a family of invariant curved transverse surfaces*, given as


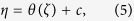


where 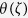 can be called the shape function and 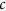 is an arbitrary constant. This means that *if we bend the originally straight transverse coordinate according to the shape of the curved surfaces by choosing a new longitudinal variable*:


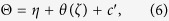


then Taylor dispersion becomes a real one-dimensional process:


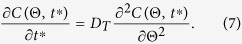


This equation can be compared with Eq. (1) which governs ***only*** the transverse mean concentration: there is not any restriction (as the mathematical operation of cross-sectional average) required for the concentration *C* in Eq. (7). In Eq. (6), the constant 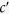 is related to the initial condition of the problem and ensures that the centroid of the solute cloud remains at the position of 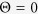, as revealed by Eq. (7). The fact that the initial condition only affects the process in such a way, actually illustrates the independence of the spatial pattern on the initial condition of the transport.

### Determination of the shape function θ

The fundamental solution of Eq. (7) is well-known:


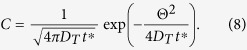


Mathematically, in this paper we refer to Taylor dispersion with reference to flat cross-sections by the {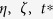}system, while we refer to the curved transverse surfaces by the {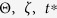} system. We can transform Eq. (8) back into the {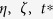} system to obtain the multi-dimensional concentration distribution:


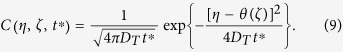


Since the transverse pattern of concentration distribution is not affected by initial condition, here we can set 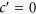, corresponding to the instantaneous and uniform initial release of solute at 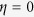 for the laminar tube flow, which is identical to the case considered by Wu and Chen^9^:


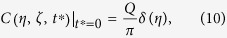


where *Q* is the released mass, and 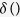 is Dirac delta function.

Expanding Eq. (9) into a Taylor series results in


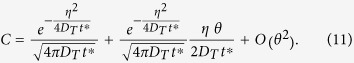


It has been shown^29^ that for a solute transport process governed by the convection-diffusion equation:


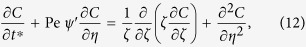


and with boundary condition


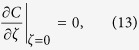


an asymptotic solution for the concentration can be expressed as^29^


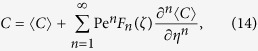


where the mean concentration is approximated as


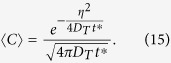


In Eq. (12), the velocity deviation


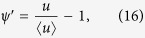


and for the present case of laminar tube flow,


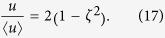


Considering Eq. (14), the cross-sectional average of Eq. (12) can be expressed as


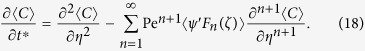


After substituting Eq. (14) into Eq. (12), and then eliminating 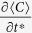 by Eq. (18), the coefficients of 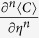 in the resulting equation should all be zero; for the term 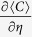, it is required that


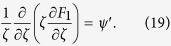


Substituting Eq. (14) into Eq. (13) results in a boundary condition for *F*_*1*_


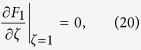


and by applying a cross-sectional average to Eq. (14) we get


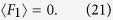


Results for *F*_*1*_ as presented in Eqs (19, 20, 21) had been deduced by different methods in previous studies^9^,^18^,^29^,^32^.

Consider *n* = 1, Eq. (14) becomes


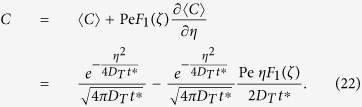


Finally, by comparing Eqs (11) and (22), the shape function *θ* can be determined as


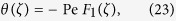


From Eqs (19, 20, 21) and (23), the shape function *θ* (or the transverse pattern of the spatial concentration distribution) is found to be directly related to a balance between the two key elements of Taylor dispersion: the shear effect and the effect of transverse diffusion. For the present case, solving Eqs (19, 20, 21) gives


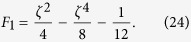


In fact, the existence of the constant 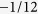 at the right hand side of the above equation will not contribute to the transverse pattern of the concentration distribution because, in Eq. (8), an arbitrary constant in 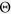 will only affect the longitudinal displacement of the solute cloud. As the transverse pattern of the distribution is determined by the terms containing 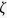 at the right hand side of Eq. (24), according to Eq. (23), the shape function 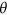 can be defined for laminar tube flow as


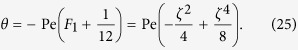


**Figure 1:**
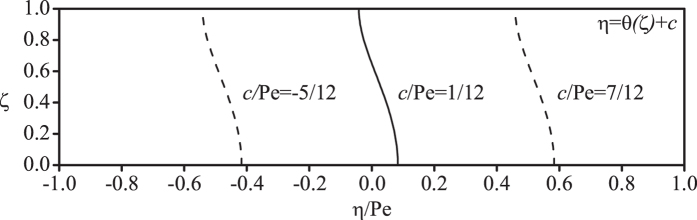
The family of curved transverse surfaces with different *c*.

In Fig. 1 we illustrate the family of curved transverse surfaces with different values of the constant *c*. Here, the special value of 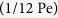 indicates that the curved transverse surface is located at the centroid of the solute cloud.

To date, we have only considered a first estimation for the spatial pattern of the concentration distribution. However, there are still effects of the higher-order terms (as appeared in Eq. (14), or the higher-order concentration modifications in our previously performed two-scale perturbation analysis^9^) that must be taken into account. The key issue on how the transverse concentration will become uniform across the family of the curved surfaces during its characteristic time-scale remains to be determined. In this paper, these concerns are addressed by resorting to the previously obtained analytical solution for the multi-dimensional concentration distribution and using numerical simulation.

### Transition to Taylor dispersion: uniform concentration across the curved surfaces

The approach to the longitudinal normality of the mean concentration^18^ has been traditionally recognized as a sign of the transition to Taylor dispersion regime. Here, we chose a new angle based on the approach to uniform concentration across the family of curved transverse surfaces.

As is known, the cause of Taylor dispersion is attributed to the combined action of flow shear and transverse diffusion^1^: in the Taylor dispersion regime, there is a balance between the shear effect (the right hand side of the Eq. (19)) and the effect of transverse diffusion (the left hand side of the Eq. (19)), which results in an invariant transverse concentration pattern, as characterized by the shape function *θ* and expressed by Eq. (23). Compared with the existing indicator for establishing Taylor dispersion based on the formation of the longitudinal Gaussian distribution, the advantages for the presently considered indicator are that: 1) the uniform concentration across the curved surfaces can be evaluated much easier and 2) it reveals the nature of Taylor dispersion.

In the previous paper^9^, the analytical solution for the complete spatial concentration distribution with modifications on the zeroth-order concentration up to the third-order was deduced to be


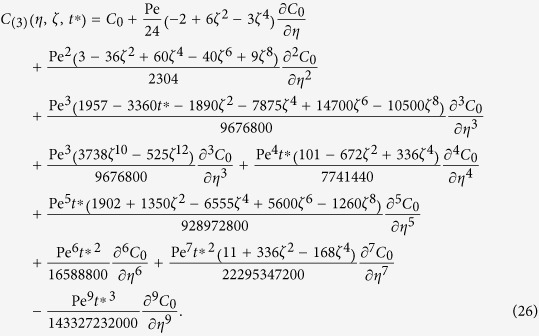


It has already been shown that Eq. (26) is consistent in form with Eq. (14)^9^. For the so-called “long time approximation”, Eq. (26) by two-scale perturbation analysis^9^ is identical to the result that can be obtained by the method of center manifold^33^,^34^, which provides a systematic and rigorous approach to calculate successive approximations. Analytical solutions for the mean concentration distribution (for example, cross-sectional average of Eq. (26)) obtained by the two methods can all be expressed as the solution of the “generalized dispersion model” 29 in the following form


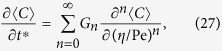


where *G*_*n*_ are constants. As *n* increases, the approximations accurately reveal the exact concentration moments as 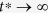, and the well-known example is its capability of capturing the variance deficit, which is a small term in the second order moment but cannot be revealed by the classic Taylor dispersion model. However, since *G*_*n*_ are constants instead of functions of time, the solutions fail at an early time of the concentration transport, as indicated by Gill and Sankarasubramanian^29^, and also in some recent comparisons between results deduced by both the Liapunov-Schmidt technique of bifurcation theory^30^ and the method of center manifold^33^,^34^.

Despite the numerous studies focusing on mean concentration, the multi-dimensional concentration distribution has received less attention. In a previous study^9^, it is also the case that only comparisons for the mean concentration have been performed for the obtained analytical (cross-sectional average of Eq. (26)) and existing numerical results. Up to now, to what extent the analytical solution Eq. (26) can give a good prediction for the real concentration distribution remains unknown. Thus, for the present exploration, we need to figure out 1) what is the concentration distribution for an early time (for example, t* = 0.2), at which Eq. (26) cannot give a good prediction (it has been shown^9^ that even the mean concentration distribution was not able to be appropriately predicted at this time); 2) at a typical time when Taylor dispersion is believed to be well established (for example, t* = 1.0), how well can Eq. (26) predict the multi-dimensional concentration distribution. These questions drove the present work, and are addressed through numerical simulations presented in the next section.

To analytically evaluate the uniform concentration across the curved transverse surfaces, a direct means is to choose a curved transverse coordinate referring to *θ*, which then results in the uniform concentration across the “flat” cross-sections under the new coordinate system. Thus, we introduce the following transformation according to Eq. (6):


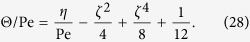


Hence, we use the constant 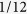 here only to adjust the longitudinal position of the resulted concentration cloud, fixing its centroid at 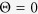. However, it does not necessarily have to be in this value for the intended evaluation because an arbitrary constant will not affect the transverse concentration distribution pattern.

The transformed analytical solution Eq. (26) in the {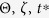} system is


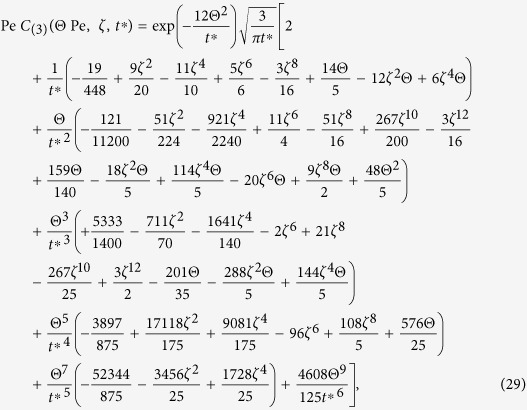


which is presented in this form to indicate that the concentration distribution can be independent of the Peclet number Pe in the system of 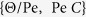 (we have neglected the insignificant longitudinal molecular diffusion effect because 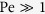 is usually the case for practical purposes), as illustrated in some later figures.

As a result, it is possible to consider the transverse concentration difference under the curved coordinate by a new indicator ***concentration difference percentage***, which is similar to that defined by Wu and Chen^9^ under the straight coordinate:


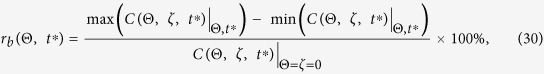


which is the ratio of the maximum concentration difference to the reference concentration for a given transverse surface at a given time.

In the next section, we show that mixing across the family of curved surfaces can be regarded as complete in the time scale of 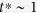, which is consistent with that for the validity of Taylor dispersion model. This means that our results are built on the well-known foundation of the establishment of Taylor dispersion: as long as the transport process enters the Taylor dispersion regime, the concentration will be uniform across such surfaces. It is vital in demonstrating some properties of the family of invariant curved transverse surfaces, one of which is that it will not be affected by the initial condition of the release. For example, a point concentration release results in a unique longitudinal Gaussian distribution of the mean concentration (not considering the longitudinal displacement of the centroid of the concentration cloud), no matter where the source is placed (at any transverse position), and the case of continuous release can be seen as the superposition of the cases of instantaneous release.

Since Taylor dispersion is an asymptotic process for the cross-sectional mean concentration to gradually approach a Gaussian distribution, traditionally we assign a time scale of t*∼1 for the transport process to enter the Taylor regime. This is partly because the degree for the real concentration distribution to approach Gaussian is hard to quantify. Now we find that during Taylor dispersion the concentration will be uniformly distributed across a family of curved surfaces, inversely, we can use this as a new criterion for entering Taylor regime, which would be much better to apply than the previous criterion focusing on the approach to Gaussian distribution. So for the first time, we can quantitatively define a threshold for such a uniformity across the curved surfaces, and we choose the value of the maximum concentration difference for the classical case (laminar tube flow) at the typical time of t* = 1 (which appears to be about 3% in the later analysis) to be the criterion.

Regarding the family of invariant curved transverse surfaces, their specific shape may vary due to different cross-sectional shapes of the confined flow region and velocity distribution (laminar or turbulent, steady or unsteady); however, there will always be such surfaces for a given flow configuration. According to the previous analysis in the paper, it is easy to find that in a general form, the function *F*_*1*_ needed for the shape function *θ* is governed by


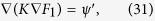



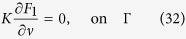



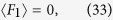


where *K* denotes the “diffusion coefficient”, which may be a function of the transverse coordinates; 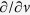 denotes differentiation along the normal to the boundary 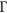; and the angle brackets 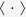 denote the properly defined transverse average operation. Thus, the family of invariant curved transverse surfaces can still be described by Eq. (5). It should be noted that Eqs (31, 32, 33) as well as Eqs (19, 20, 21) are not new, but only constitute essential elements of the crux relation (Eqs (5) and (23)) between what is known (*F*_*1*_), and what is unknown (the family of the curved surfaces) in the paper. This can also be seen as a mathematical illustration of the fact that our finding is exactly based on the established theory of Taylor dispersion.

## Results and Discussions

We performed numerical simulations for scalar transport in laminar tube flow to obtain an idea about the real concentration distribution and its evolution during the transition to the Taylor dispersion regime. The numerical efforts were made based on the finite element method (COMSOL, http://www.comsol.com/) for the convection-diffusion equation with its boundary condition as given by Eqs (12) and (13).

We can rewrite Eq. (12) as


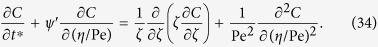


where the combination 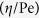 can be seen as a new longitudinal variable. For the Taylor dispersion process, the molecular diffusion effect can usually be neglected for practical purposes because of a large value of Pe^9^,^29^ (for example, at the order of 10^3^ or 10^4^), then the last term at the right hand side of Eq. (34) can be discarded to get:


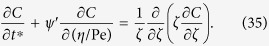


Notice that there are no additional independent parameters in Eq. (35), thus reducing the computational cost of the simulation. The initial release is considered to be of unit mass


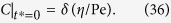


Since Eq. (35) indicates a transport process observed for a longitudinally moving coordinate system with the mean flow velocity, during the entire process the centroid of the solute cloud is centered at 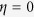. We carefully chose the computational region to ensure that at the end of each selected case (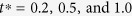, respectively), the solute cloud was properly captured within the whole region. Therefore, the mesh size of 200 × 200 was found sufficient for all three cases, validated by additional computations with increased mesh sizes. For the initial condition Eq. (36), we chose the central two columns of grids around 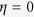, that is, altogether 400 grids to have a uniform concentration while the other grids are with zero concentration. This treatment was also found capable of giving the correct initial condition for the chosen cases.

By a first glance of the results, we can have some qualitative impression on the concentration distribution differences, either across the flat plane or the deduced curved transverse surfaces. The quantitative descriptions will be provided in later contents associated with the defined indicators for the differences.

**Figure 2:**
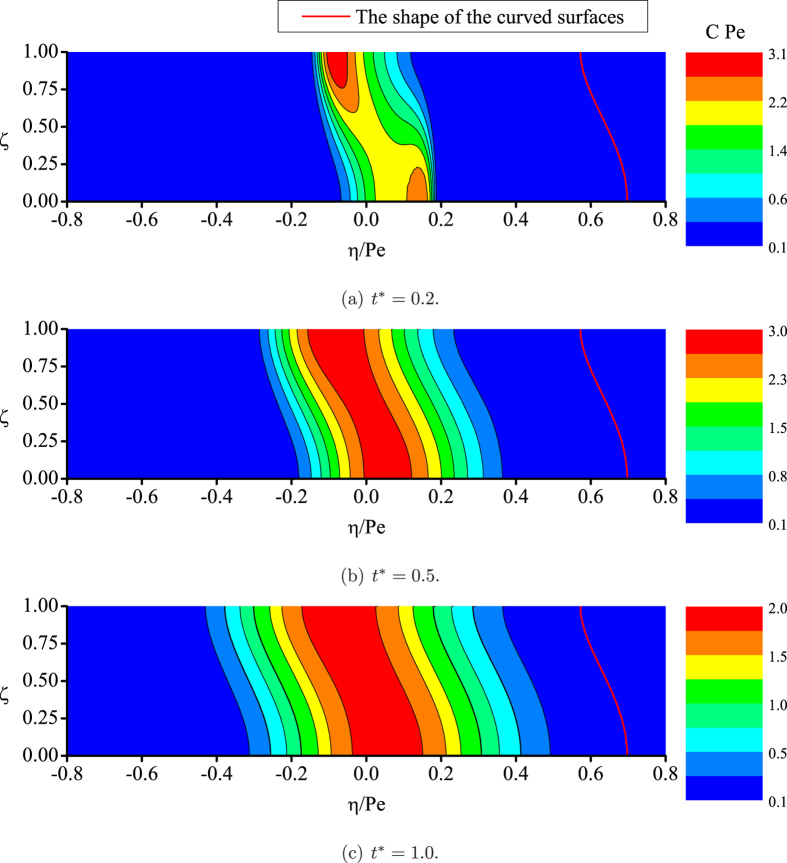
Numerical results for scalar transport in laminar tube flow: the concentration contours.

In Fig. 2(a), it is obvious at this early stage that the mixing is insufficient, either across the flat cross-sections or the curved transverse surfaces. It is the crossover region in the neighborhood of 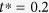 for different mechanisms to dominate the scalar transport process^19^: usually at an earlier time the process is dominated by convection, and at the later time it generally falls into the Taylor dispersion regime, although with certain non-Gaussian features (such as asymmetry) remained to some extent for the longitudinal concentration distribution. At a later time of 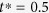, from the contours we find the uniformity of the concentration distribution across the curved surfaces is relatively achieved, while the radial concentration difference remains high. Generally, the mixing across the curved surfaces for the downstream (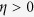) cloud is better, and a less uniform region appears at the vicinity of tube wall for the upstream cloud.

Figure 2(c) shows that the mixing across the curved surfaces can already be regarded as complete at 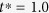, despite the noticeable longitudinal asymmetry between upstream and downstream extents of the cloud. Thus, achieving a uniformly distributed concentration across the curved surfaces can be observed to be a new indication of the transition to the Taylor dispersion regime for scalar transport. On the other hand, since it is commonly believed that the non-Gaussian properties for the mean concentration distribution generally dampen out at this time^18^, the sole time scale of 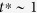 is sufficient to characterize the transition; and the two respective basic processes for the longitudinal and transverse concentration evolution can finally be regarded as one via observations with reference to the family of curved transverse surfaces.

**Figure 3:**
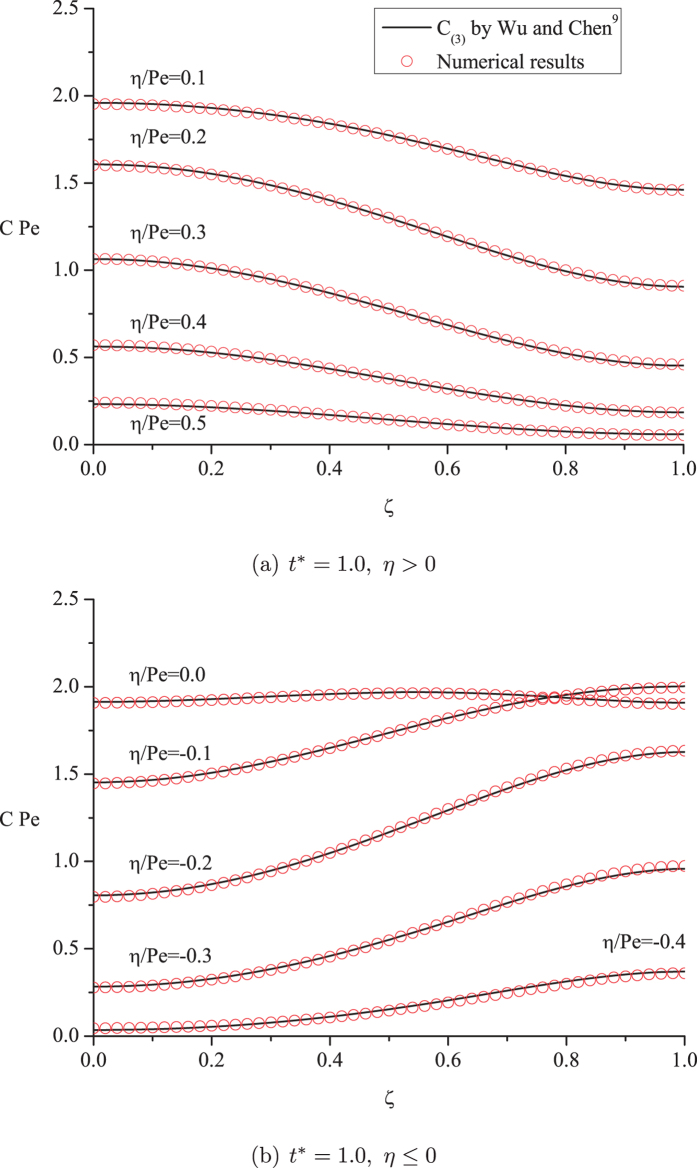
Concentration distributions across some flat cross-sections of the cloud.

By some flat transverse cross-sections, in Fig. 3, we provide further comparisons between the results of the presently performed numerical simulations and the analytical solution by Wu and Chen^9^. As we mentioned, the performance of Eq. (26) was not yet fully understood in the previous paper: For the first time, we numerically verified the predictions of 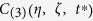 for the complete spatial concentration distribution, and found excellent agreement with the numerical results. Thus 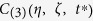 is referred to as the “real concentration distribution” for the Taylor dispersion regime from here on.

In Fig. 3, the concentration is shown to deviate to a large degree from a uniform distribution across the flat cross-sections of the cloud. Importantly, the figure reveals the complicated patterns of the concentration distribution observed in a reference frame that is not so appropriate: from the flat cross-sections, it is difficult to achieve some sound idea regarding the characteristics for the concentration distribution in the solute cloud. For example, it is unexpected at first glance that “the maximum transverse concentration difference is found on some intermediate cross-section, while the minimum is near the center and the edges of the cloud” 9.

**Figure 4:**
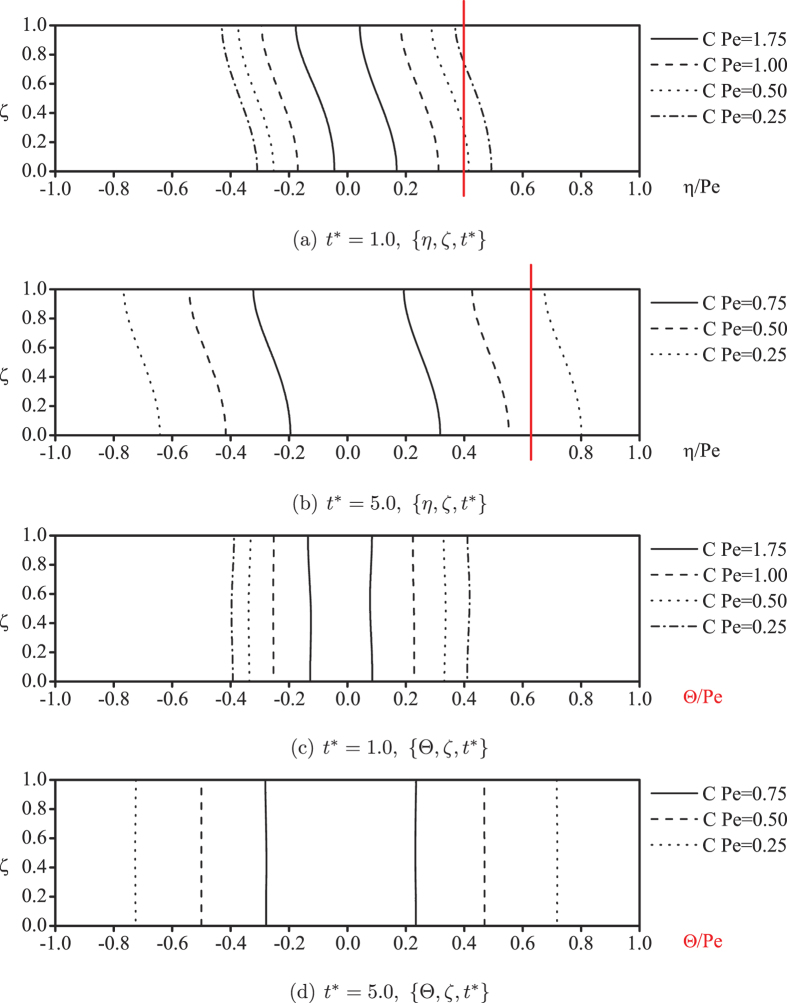
Concentration contours with reference to the flat and curved transverse surfaces, respectively.

The uniform concentration distribution is more gradual across the flat cross-sections. This can be observed in Fig. 4(a,b) on the reduction of the cross-sectional concentration difference: even for long times, as 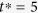, the uniformity of the concentration is not so well established transversely. Quantitatively for the maximum concentration of the cloud, the maximum concentration difference percentage was found to be close to 20%^9^. The decrease of the transverse concentration difference can be observed as the consequence of the separation of the concentration contours, as demonstrated by a flat cross-section in red between the same contours of 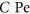 = 0.25 and 0.50 in Fig. 4(a,b), which is actually caused by the decrease of the longitudinal concentration gradient. An important feature found in these two subfigures is that the shape of the contours remains unchanged for different concentrations and times, which indicates perfect reference surfaces for describing the pattern of the evolution of the concentration.

To generally understand the process for the “real concentration distribution” to achieve uniformity across the curved transverse surfaces, we refer to 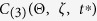 by Eq. (29), and the concentration contours are plotted in Fig. 4(c,d). From the excellent results, the transverse concentration is found to be nearly uniformly distributed in the 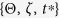 system at 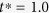, which is much better than the slowly asymptotic uniformity achieved in the 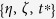 system, as shown by Fig. 3 at the same time. In Fig. 4(d), the uniformity is further improved with straight lines to represent the contours in the figure in comparison with the aforementioned 20% difference in the maximum concentration of the cloud in the {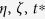} system.

**Figure 5:**
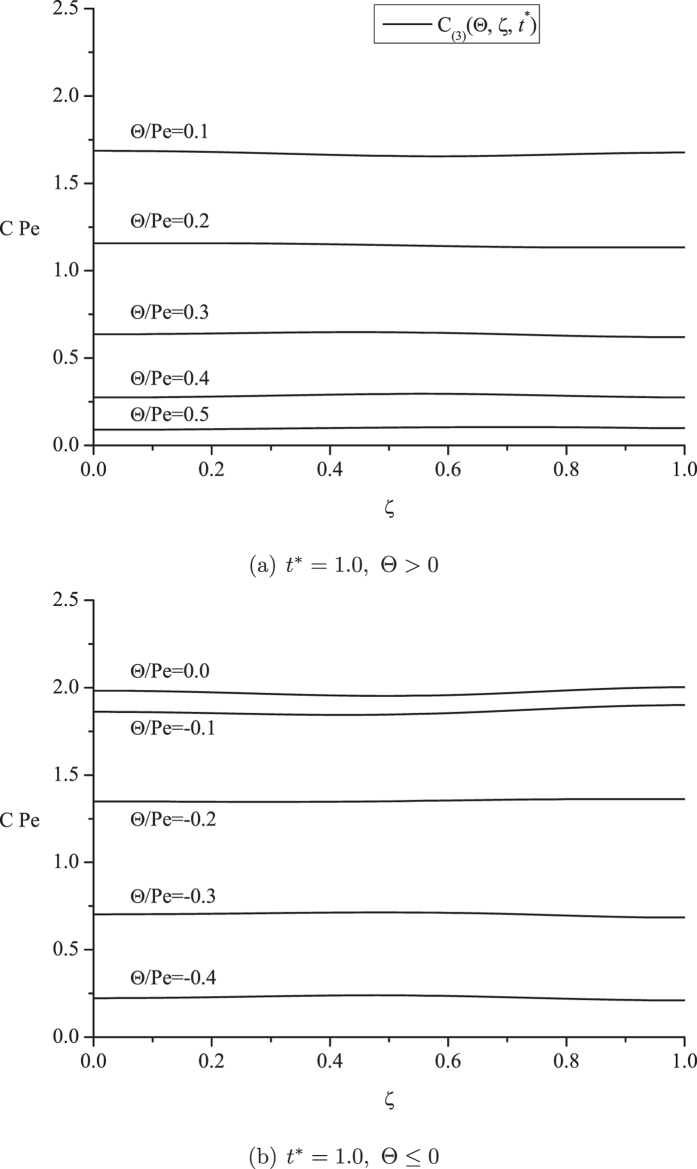
Concentration distributions at different longitudinal positions across the curved transverse surfaces.

Figure 5 gives detailed information for the concentration distribution across the curved transverse surfaces at different longitudinal positions. Instead of revealing a large concentration variation across the flat cross-sections, as shown in Fig. 3, the concentration distributions given in Fig. 5 are nearly independent of the curved transverse coordinate.

**Figure 6:**
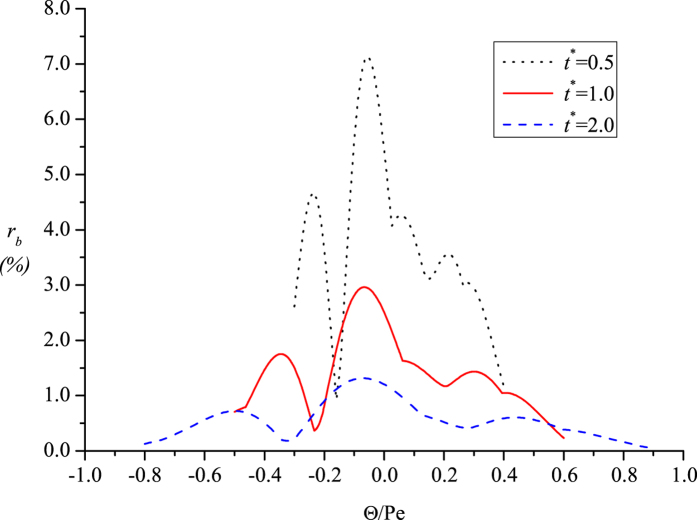
Longitudinal distribution of the concentration difference percentage across the curved transverse surfaces at different times.

To exactly reveal the longitudinal distribution of the concentration difference and the process for the real concentration distribution to achieve uniformity across the curved transverse surfaces, we refer to 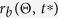 by Eq. (30) in Fig. 6. Because we generally understand that the uniformity is achieved at the time scale of *R*^*2*^*/D*, three typical dimensionless times of t* = 0.5, 1.0 and 2.0 are chosen.

From the curves in the figure, the concentration difference is shown to reach the maximum in the vicinity of longitudinal center of the cloud. A relatively more intense fluctuation of the difference percentage exists for the upstream half of the concentration cloud than that for the downstream half. At longer times, the maximum difference percentage is found to decrease from ∼7% to ∼1%, confirming the excellent uniformity that is achieved across the curved transverse surfaces at these times. For the typical time of t* = 1.0, a maximum difference percentage of ∼3% is found across the curved surfaces, which can be compared with the previous value of nearly 50% across the flat cross-sections at the same time. To achieve transverse uniformity to the same degree (∼3% by difference percentage) in the {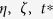} system, two orders of magnitude longer times (t* = ∼100) are required^9^.

The present findings on the uniform concentration across the curved surfaces also enable the quantitative definition of a new criterion for entering Taylor dispersion, and the 3% maximum concentration difference for the typical case can be adopted as a threshold. This can be compared with the traditionally considered criterion focusing on the approach to longitudinal normality for the mean concentration.

## Conclusions

The main finding of the present paper is the characterization of the pattern of the complete spatial concentration distribution. For scalar transport in laminar tube flow, at the time-scale of *R*^*2*^/*D*, the transverse concentration approaches a uniform concentration across a family of invariant curved surfaces, instead of the flat cross-sections in the traditional view. In addition, the previously discussed different types of transverse concentration variations are shown to be the result of observation in a “flat” reference frame, which is not appropriate for the process.

During the analytical analysis, the family of curved surfaces for the uniform concentration is shown to be caused by the final balance between flow shear and transverse diffusion, which are the key elements for the concept of Taylor dispersion. Thus, although the present study is based on the analysis of the simple laminar tube flow, the conclusion regarding the curved transverse surfaces can be valid for Taylor dispersion in more complicated flows (turbulent, unsteady, etc.) with different cross-sectional shapes.

Observing the approach to a uniform concentration across the curved surfaces is a new angle for the transition to the Taylor dispersion regime, and its advantages are that: 1) the uniform concentration distribution can be evaluated much easier than the longitudinal normality and 2) it reveals the nature of Taylor dispersion. The evaluation in the paper is accomplished by transforming the straight transverse coordinate for the previously obtained analytical solution into the curved one according to the deduced shape function. An indicator for the concentration difference across the curved surfaces is defined under the curved coordinate by analogy to that under the straight one; it reveals a maximum difference percentage of ∼3% across the curved surfaces compared with the previous value of nearly 50% across the flat cross-sections at the same time of t* = 1.0. To achieve transverse uniformity to the same degree (∼3% by concentration difference percentage) at the same time across the flat cross-sections, two orders of magnitude longer times (t* = ∼100) are required.

## Additional Information

**How to cite this article**: Wu, Z. *et al.* On spatial pattern of concentration distribution for Taylor dispersion process. *Sci. Rep.* **6**, 20556; doi: 10.1038/srep20556 (2016).
